# Risk Factors for MDR and XDR-TB in a Tertiary Referral Hospital in India

**DOI:** 10.1371/journal.pone.0009527

**Published:** 2010-03-04

**Authors:** V. Balaji, Peter Daley, Alok Azad Anand, Thambu Sudarsanam, Joy Sarojini Michael, Rani Diana Sahni, Poorvi Chordia, Ige Abraham George, Kurien Thomas, Alka Ganesh, K. R. John, Dilip Mathai

**Affiliations:** 1 Department of Microbiology, Christian Medical College Vellore, Vellore, Tamil Nadu, India; 2 Department of Medicine, Christian Medical College Vellore, Vellore, Tamil Nadu, India; 3 Tufts University School of Medicine, Boston, Massachusetts, United States of America; 4 Department of Community Medicine, Christian Medical College Vellore, Vellore, Tamil Nadu, India; U.S. Naval Medical Center Research Detachment, Peru

## Abstract

**Background:**

India has a high burden of drug resistant TB, although there are few data on XDR-TB. Although XDR-TB has existed previously in India, the definition has not been widely applied, and surveillance using second line drug susceptibility testing has not been performed. Our objective was to analyze clinical and demographic risk factors associated with isolation of MDR and XDR TB as compared to susceptible controls, at a tertiary center.

**Methodology/Findings:**

Retrospective chart review based on positive cultures isolated in a high volume mycobacteriology laboratory between 2002 and 2007. 47 XDR, 30 MDR and 117 susceptible controls were examined. Drug resistant cases were less likely to be extrapulmonary, and had received more previous treatment regimens. Significant risk factors for XDR-TB included residence outside the local state (OR 7.43, 3.07-18.0) and care costs subsidized (OR 0.23, 0.097-0.54) in bivariate analysis and previous use of a fluoroquinolone and injectable agent (other than streptomycin) (OR 7.00, 95% C.I. 1.14-43.03) and an initial treatment regimen which did not follow national guidelines (OR 5.68, 1.24-25.96) in multivariate analysis. Cavitation and HIV did not influence drug resistance.

**Conclusions/Significance:**

There is significant selection bias in the sample available. Selection pressure from previous treatment and an inadequate initial regimen increases risk of drug resistance. Local patients and those requiring financial subsidies may be at lower risk of XDR-TB.

## Introduction

India has the greatest burden of Tuberculosis (TB) disease in the world, with 1.8 million new cases annually and an estimated prevalence of 3.8 million bacteriologically proven cases in 2000.[1] Annual loss to the country's economy due to TB is greater than US$ 3 billion.[2]

Multidrug resistant TB (MDR-TB) is defined as resistance to the two most important first-line drug treatments, isoniazid and rifampicin. Extensively drug resistant TB (XDR-TB) is resistant to these first line agents, as well as to at least one fluoroquinolone and at least one injectable agent.[3] This phenotype emerges from MDR-TB, with the acquisition of further drug resistance mutations, and was first described in the United States[4], followed by the Tugela Ferry outbreak.[5] XDR-TB is associated with a significantly worse clinical outcome,[6], [7] and risk factors for poor treatment response have been defined.[8] MDR and XDR represent distinct phenotypes and are considered separately in this paper and other publications. Primary drug resistance is defined as cases which have not be previously treated, and secondary drug resistance occurs in pre-treated patients.

The objective of our study was to describe the clinical and demographic risk factors associated with the isolation of XDR-TB in a tertiary hospital laboratory in South India. We surveyed drug susceptibility test (DST) results between 2002 and 2007 and identified isolates meeting the criteria for MDR and XDR-TB. We retrospectively reviewed these cases and compared them to controls with susceptible disease.

## Methods

### Ethics Statement

The study was approved by the ethics review board of the Christian Medical College Vellore in February 2008. Patient consent was not obtained because data were analyzed anonymously.

### DST Testing

All DST was performed by the mycobacteriology section of the Clinical Microbiology Department at the Christian Medical College Vellore, which is externally accredited by the Revised National Tuberculosis Control Program and the Central Tuberculosis Division, Ministry of Health, Government of India. DST for first-line (isoniazid, rifampicin, ethambutol and streptomycin) and second line (ciprofloxacin, ethionamide, capreomycin and cycloserine) drugs was performed by absolute concentration method (MIC) for all drugs except streptomycin, in which the resistance ratio method was used. During 2007, the DST method for first line drugs was changed to the 1% proportion method. Drugs were procured from Sigma (USA), and for each batch of DST, a sensitive strain of H37Rv was used as a control.

### Case Definitions

Results for indicator drugs Isoniazid, Rifampicin, Ciprofloxacin (fluoroquinolone class) and Capreomycin (injectable class) were used in the definition. Isolates resistant to both Isoniazid and Rifampicin only were defined as MDR, and isolates resistant to all four of these drugs were defined as XDR.

### Case Inclusion

**Figure 1:**
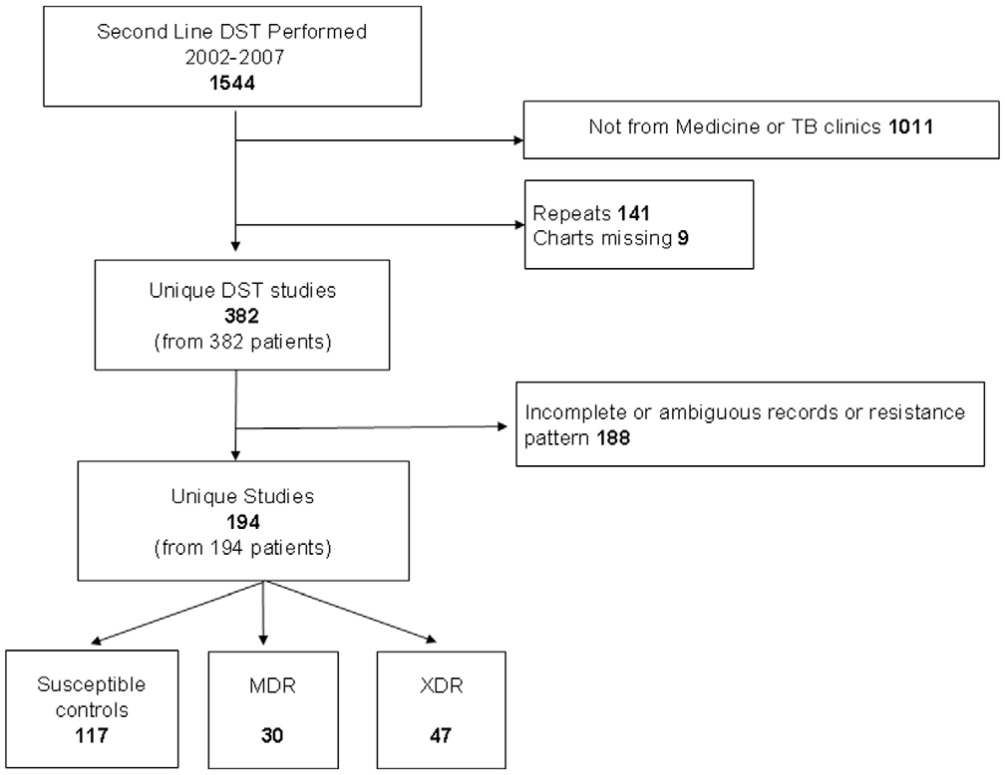
Recruitment.

The Mycobacteriology laboratory DST registers between 2002 and 2007 were reviewed. Inclusion in the study was limited to specimens growing *Mycobacterium tuberculosis* which were tested for first and second line DST by request of the physician, and which were available for our evaluation (ordered from the department of Medicine or from TB clinic). After isolates were identified from the laboratory register, clinical information was sought. See Figure 1 for a summary of recruitment. There were no standard criteria for referral for DST.

Between 2002 and 2006, all available DST results were considered. Control isolates were susceptible to all tested drugs. In 2007, the number of DST studies performed was much higher, so for this year, susceptible controls were selected randomly (Microsoft Excel), in a ratio of 3 susceptible controls for each MDR and XDR case.

### Population

The Christian Medical College Vellore is a 2200 bed tertiary referral hospital in Vellore, Tamil Nadu. Tamil Nadu has a TB case detection rate of 131/100,000 per year.[2] Most TB patients assessed have been previously seen or treated. Culture and DST is not paid for by the government TB control program in India.

### Analysis

Predefined risk factors associated with drug resistance were included in a case report form, which was piloted on 10 patient charts. Clinical, demographic and WHO defined[9] risk factors were included based on previous literature, and drug treatment history was extracted. The following risk factors were examined: HIV infection[10] (tested in 134/203 (66%) cases), socioeconomic status[11] (as approximated by the provision of subsidy for the cost of care), gender, age category, cigarette use, alcohol use[1], site of TB disease[1], diabetes[1], residence outside of the state (referral patients) [12], cavitation on CXR[11], number of different previous treatment regimens[13], previous use of injectable agents and fluoroquinolones[13] and adequacy of initial treatment (defined as whether or not the initial treatment regimen followed the regimen outlined by the national treatment program for new smear positive TB patients[2]). Descriptors and risk factors were compared using independent samples T-test. Bivariate and multivariable logistic regression was performed to compare MDR and XDR cases to susceptible cases (SPSS 13.0).

## Results

1544 cultures with first and second line DST were available between 2002 and 2007 (Figure 1). Among these, 532 were ordered by Medicine units or TB clinic, 382 of which were unique (one specimen per patient). Of these, 188 had incomplete clinical information available, or did not have complete DST and thus could not be assigned to one resistance category, leaving 194 results available for complete analysis. Of these, 47 patients had XDR-TB and 30 had MDR-TB. The group of susceptible controls was collected by combining the entire population of susceptible cases from 2002–2006 with the randomly selected susceptible group from 2007. 117 patients were used as susceptible controls.

The 117 Susceptible cases were 20.5% females (24/117), with a mean age of 42 years (SD 14.5) (see Table 1). Twenty three were only extrapulmonary cases (20.5%). Eighty eight were tested for HIV, among which 25 were HIV infected (28.4%). Mean number of different treatment regimens prior to culture was 2.5 (SD 1.7). Thirty three had cavitation on CXR (29.2%), and 9 (8.5%) had received treatment with both an injectable agent (other than streptomycin) and a fluoroquinolone.

**Table 1:** Description of Cases.

	Susceptible	MDR	XDR
Total Cases N = 194	117	30	47
Mean age N = 194	42.7+/−14.5	37.3+/−14.2 (p = 0.066 vs Susc)	37.0+/−12.5 (p = 0.020 vs Susc)
Female N = 194	24/117 (20.5%)	8/30 (26.7%) (p = 0.47 vs Susc)	14/47 (29.8%) (p = 0.21 vs Susc)
Only Extrapulmonary TB N = 190	23/113 (20.5%)	1/30 (3.3%) (p = 0.002 vs Susc)	3/29 (10.3%) (p = 0.001 vs Susc)
HIV Infected* N = 131	25/88 (28.4%)	1/14 (7.2%) (p = 0.092 vs Susc)	3/47 (6.4%) (p = 0.049 vs Susc)
Mean number of difference treatment regimens before culture N = 183	2.5+/−1.7	4.7+/−1.7 (p<0.001 vs Susc)	4.2+/−2.0 (p<0.001 vs Susc)
Previous treatment with a quinolone and injectable agent (other than streptomycin) N = 181	9/106 (8.5%)	19/30 (63.3%) (p<0.001 vs Susc)	28/45 (62.2%) (p<0.001 vs Susc)
Cavity present on CXR N = 185	33/113 (29.2%)	13/31 (41.9%) (p = 0.17 vs Susc)	22/47 (46.8%) (p = 0.048 vs Susc)

*More than 20% data unavailable.

The 30 MDR-TB cases were 26.7% females (8/30), with a mean age of 37.3 years (SD 14.2). Only one was extrapulmonary disease (3.3%, p = 0.002 vs susceptibles). Fourteen were tested for HIV, among which 1 was HIV infected (7.2%, p = 0.092 vs susceptibles). Mean number of different treatment regimens prior to culture was 4.7 (p<0.001 vs susceptibles). Thirteen (41.9%, p = 0.17 vs susceptibles) had cavitation on CXR and 19 (63.3%, p<0.001 vs susceptibles) had received treatment with both an injectable agent (other than streptomycin) and a fluoroquinolone. All cases had been previously treated at least once.

The 47 XDR-TB cases were 29.2% females (14/47), with a mean age of 37.0 years (SD 12.5). Only 3 were extrapulmonary cases (10.3%, p = 0.001 vs susceptibles). All were tested for HIV, among which 3 were infected (6.4%, p = 0.049 vs susceptibles). Mean number of different treatment regimens prior to culture was 4.2 (p<0.001 vs susceptibles). Twenty-two (46.8%, p = 0.048 vs susceptibles) had cavitation on CXR and 28 (62.2%, p<0.001 vs susceptibles) had received treatment with both an injectable agent (other than streptomycin) and a fluoroquinolone. All cases had been previously treated at least once.

In the bivariate logistic regression analysis, three variables were associated with MDR as compared to susceptible TB (see Table 2). Previous treatment with an injectable and fluoroquinolone was strongly associated with MDR (OR 18.62, 95% C.I. 6.78–51.06). Smoking was negatively associated with MDR (OR 0.23, 95% C.I. 0.063–0.81) as was alcohol use (OR 0.11, 95% C.I. 0.014–0.82). Residence outside the state of Tamil Nadu demonstrated a trend towards association (OR 2.25, 95% C.I. 0.98–5.15).

**Table 2:** Bivariate Analysis: Risk factors for MDR.

Risk factor	Odds Ratio for MDR	95% C.I.
Age Category		
Age <21	reference	
Age 21–30	0.78	0.12–5.34
Age 31–40	0.12	0.014–1.04
Age 41–50	0.60	0.079–4.54
Age >50	0.27	0.038–1.92
Female Sex	1.41	0.56–3.56
HIV infection*	0.19	0.024–1.56
Smoking	0.23	0.063–0.81
Alcohol use	0.11	0.014–0.82
Diabetes*	0.90	0.33–2.45
Residence outside Tamil Nadu state	2.25	0.98–5.15
Care costs subsidized	0.79	0.33–1.89
Initial treatment regimen did not follow national guidelines	1.6	0.67–4.00
Previous treatment with a fluoroquinolone and an injectable agent (other than streptomycin)	18.62	6.78–51.06
CXR shows cavity	1.78	0.78–4.09
Number of different treatment regimens	1.89	1.45–2.45
Extrapulmonary TB	0.14	0.017–1.04

*More than 20% data unavailable.

A multivariable regression was created using the following variables, considered either statistically or clinically significant: smoking, alcohol, HIV, residence outside Tamil Nadu state, extrapulmonary TB, costs of care subsidy, cavitation on CXR, number of previous treatment regimens, previous treatment with a fluoroquinolone and an injectable agent, and if first drug treatment regimen followed national guidelines (see Table 3) None of these were significantly associated with MDR-TB.

**Table 3:** Bivariate Analysis: Risk factors for XDR.

Risk factor	Odds Ratio for XDR	95% C.I.
Age Category (years)		
Age <21	reference	
Age 21–30	1.04	0.16–6.97
Age 31–40	0.49	0.072–3.27
Age 41–50	0.90	0.13–6.46
Age >50	0.31	0.044–2.15
Female Sex	1.64	0.76–3.55
HIV infection*	0.29	0.081–1.05
Smoking	0.43	0.18–1.03
Alcohol use	0.21	0.060–0.74
Diabetes*	0.82	0.35–1.91
Residence outside Tamil Nadu state	7.43	3.07–18.0
Care costs subsidized	0.23	0.097–0.54
Initial treatment regimen did not follow national guidelines	5.85	2.53–13.52
Previous treatment with a fluoroquinolone and an injectable agent (other than streptomycin)	17.75	7.14–44.14
CXR shows cavity	1.78	0.78–4.09
Number of different treatment regimens	1.60	1.29–1.98
Extrapulmonary TB	0.27	0.076–0.94

*More than 20% data unavailable.

In the bivariate logistic regression analysis, six variables were associated with XDR as compared to susceptible TB (see Table 3): previous treatment with an injectable and fluoroquinolone (OR 17.75, 95% C.I. 7.14–44.14), residence outside the state of Tamil Nadu (OR 7.43. 95% C.I. 3.07–18.00) and an initial treatment regimen that did not follow national guidelines (OR 5.85, 95% C.I. 2.53–13.52) were positively associated with XDR. Alcohol use (OR 0.21, 95% C.I. 0.060–0.74), extrapulmonary TB (OR 0.27, 95% C.I. 0.076–0.94) and cost of care subsidized (OR 0.23, 95% C.I. 0.097–0.54) were negatively associated with XDR.

A multivariable regression was created using the same variables as above (see Table 4). Two variables were independently associated with XDR-TB: previous treatment with a fluoroquinolone and an injectable agent (other than streptomycin) (OR 7.00, 95% C.I. 1.14–43.03), and an initial treatment regimen which did not follow national guidelines (OR 5.68, 95% C.I. 1.24–25.96).

**Table 4:** Multivariate Analysis: Risk factors for XDR.

Risk Factor	Odds Ratio for XDR	95% C.I.
Previous treatment with a fluoroquinolone and an injectable agent (other than streptomycin)	7.00	1.14–43.03
Initial treatment regimen did not follow national guidelines	5.68	1.24–25.96

Including smoking, alcohol, HIV, TN state, payment, regimen 1 adequate, extrapulmonary TB, cavitary, number of regimens, previous treatment with fluoroquinolone and injectable.

Only significant associations shown.

Model based on 69 cases with complete data.

The most common deviation from national treatment guidelines was the use of correct drugs for a duration longer than six months.

## Discussion

We have undertaken a retrospective review of risk factors associated with isolation of MDR and XDR as compared to susceptible TB in a tertiary care referral center in India, the first study to do so to date. Our study population is not representative of TB patients in India due to selection bias, referral bias, and selective testing bias. For this reason, we do not report a prevalence rate of XDR-TB.

A group of 141 patients did not have enough DST data to be classified into a resistance category, and were excluded from analysis. In addition, only a single fluroquinolone and one injectable agent were tested. Thus, XDR patients infected with strains resistant to fluoroquinolones or injectable agents other than those tested could be misclassified as MDR or drug-susceptible TB. Due to our retrospective design, some of the variables examined were inconsistently recorded or missing.

Our sample size of drug resistant isolates is small, although we were able to screen a large number of isolates. Second line drug susceptibility testing is not well standardized, so we cannot refer to a standard laboratory method to corroborate our fluoroquinolone or injectable susceptibility results.

We detected no primary drug resistance in our highly selected population. Primary drug resistance is known to be uncommon in India.

Nine surveys of TB drug resistance have been done in India between 1997 and 2006. Primary MDR-TB was reported in between 0.5 and 3.4% (9 studies), and secondary MDR-TB was 17.2% (1 study).[10] These incidence rates are similar to global estimates of 2.9% and 15.3% respectively. However, since retreatment cases represent 13.7% of notified cases in India, the total burden of drug resistant TB is very high, with an estimate that 110,132 new MDR-TB cases emerged in India in 2006, [10] the most cases of any country.[14]

Although XDR-TB has existed previously in India[15], the recent definition has not been widely applied, and community-based surveillance has not be performed. Second line drugs are widely available in the private sector, where most Indian TB patients seek care.[16] However, drug susceptibility testing is not widely available, although, the expectation is that there is a large burden of resistant TB that will be uncovered with the wider availability of culture laboratories proposed by the national TB control program.[1]

XDR-TB has been described from 55 countries, and in all world regions.[14] The first report of XDR-TB from India surveyed 3,904 specimens, and thirty three cases met the definition of XDR-TB, representing 8% of all MDR-TB cases.[17] Five among 68 MDR isolates from Lucknow met the definition for XDR.[18] Among 66 MDR patients on treatment, one had XDR-TB, and two developed an XDR phenotype during treatment.[19] In a fourth report, 54 HIV infected TB suspects were investigated and four met the criteria for XDR-TB, and all died with 2.6 months of diagnosis.[20]

In a prospective study of 130 MDR and 130 drug susceptible TB patients at a tertiary referral center in Delhi, a multivariate model showed poor compliance, greater than one cavity, and HLA DRB1*14 allele were associated with MDR.[11] In a prospective study of 251 TB patients in Pune, 52 MDR cases were identified, 36 (69.2%) of whom had a history of treatment default, and 11 (21.2%) of whom were relapse cases.[21] The most important reason for treatment default among MDR cases was travel (17/36), followed by adverse effects of drugs (9/36), lack of symptom relief (8/36) and cost (2/36).

We report that previous use of both a fluoroquinolone and an injectable agent (other than streptomycin) is associated with XDR, which is expected since these agents may select for resistance when used for second line TB treatment.[22] Referral patients from outside the state of Tamil Nadu may be associated with drug resistance. The referral bias of poor treatment responders from different geographical area in India to our hospital is the best explanation for this association.

Our data suggests that patients who cannot afford to pay for their TB treatment may be less associated with the isolation of XDR. One possibility for this observation is that these patients are more likely to receive first line treatment in the national TB control program (because they cannot afford private medical care), which is associated with higher adherence rates.[23] It is possible that these patients may not have been able to afford second line treatment. However, the possibility that poorer patients with XDR TB are less likely to come to the referral hospital or be fully investigated microbiologically cannot be excluded.

Our patients with drug resistant TB had received up to 10 different treatment regimens prior to submitting the positive culture. Drug resistant patients in our study had a greater number of previous regimens than susceptible patients, as has been also shown among XDR cases in Peru.[13]

Our data suggests that the an inadequate initial drug regimen may be associated with the development of XDR, but with the limitations of our design we cannot make this conclusion.

Our data suggests no association between HIV infection and drug resistance, although because only 67.5% of patients in our study were tested for HIV, we cannot make a conclusion on this. Larger studies in Latvia and Donetsk Oblast have suggested an association between HIV and MDR-TB, [10] and in the United States, HIV was significantly more common among XDR cases as compared to drug susceptible cases.[24] With an overall HIV prevalence of less than 1%, India may not yet have enough HIV cases to experience a significant overlap between the HIV and drug resistant TB epidemics. During our study period, most patients with HIV were not on antiretroviral treatment, and it is probable that co-infected patients succumbed to the disease before being identified and treated as MDR or XDR-TB.

We found that smoking and alcohol may be associated with a lower risk of MDR-TB, and alcohol use may be associated with a lower risk of XDR-TB. This finding may be biased by the loss of individuals with these risk factors due to a generally poor state of health or socio-economic status causing early death after developing resistant TB, prior to submitting a specimen for culture.

A decision must be made by a clinician to request a drug susceptibility test from a TB patient. At our center, the decision to order such testing is up to individual clinicians, based on medical as well as economic factors, as this test may not be affordable to the patient. The identification of clinical risk factors may help the clinician decide when to request the test. According to the national TB control program, DST should be performed on patients taking category II treatment who remain smear positive after the fourth month of treatment.[25] However, this strategy is a necessary balance between operational considerations and early diagnosis, and will provide delayed results for many cases with primary or acquired drug resistance.

The development of drug resistance in TB in India is the result of a complex web of biomedical, socio-cultural and behavioral interactions,[26] and the reporting of individual risk factors is an oversimplification. Health care worker ignorance, the wide misuse of TB drugs, lack of laboratory standardization and delay in laboratory results all contribute to the emergence of drug resistance.[27]
